# Improving interprofessional coordination in Dutch midwifery and obstetrics: a qualitative study

**DOI:** 10.1186/1471-2393-14-145

**Published:** 2014-04-15

**Authors:** Vera LN Schölmerich, Anke G Posthumus, Halleh Ghorashi, Adja JM Waelput, Peter Groenewegen, Semiha Denktaş

**Affiliations:** 1 Department of Obstetrics and Gynecology, Division of Obstetrics and Prenatal Medicine, Erasmus University Medical Centre, Westzeedijk 118, Rotterdam 3016 AH, The Netherlands; 2 Department of Organizational Sciences, VU University Amsterdam, De Boelelaan 1081, Amsterdam 1081 HV, The Netherlands; 3 Department of Sociology, VU University Amsterdam, De Boelelaan 1081, Amsterdam 1081 HV, The Netherlands

**Keywords:** Interprofessional coordination, Prenatal health, Antenatal health, Midwifery and obstetrics, Maternal health care, Coordination practices

## Abstract

**Background:**

Coordination between the autonomous professional groups in midwifery and obstetrics is a key debate in the Netherlands. At the same time, it remains unclear what the current coordination challenges are.

**Methods:**

To examine coordination challenges that might present a barrier to delivering optimal care, we conducted a qualitative field study focusing on midwifery and obstetric professional’s perception of coordination and on their routines. We undertook 40 interviews with 13 community midwives, 8 hospital-based midwives and 19 obstetricians (including two resident obstetricians), and conducted non-participatory observations at the worksite of these professional groups.

**Results:**

We identified challenges in terms of fragmented organizational structures, different perspectives on antenatal health and inadequate interprofessional communication. These challenges limited professionals' coordinating capacity and thereby decreased their ability to provide optimal care. We also found that pregnant women needed to compensate for suboptimal coordination between community midwives and secondary caregivers by taking on an active role in facilitating communication between these professionals.

**Conclusions:**

The communicative role that pregnant women play within coordination processes underlines the urgency to improve coordination. We recommend increasing multidisciplinary meetings and training, revising the financial reimbursement system, implementing a shared maternity notes system and decreasing the expertise gap between providers and clients. In the literature, communication by clients in support of coordination has been largely ignored. We suggest that studies include client communication as part of the coordination process.

## Background

Dutch midwifery and obstetrics distinguishes three levels of care: primary, secondary and tertiary care. Community midwives situated in neighborhood practices provide primary care, while obstetric caregivers in hospitals provide secondary and tertiary care. Community midwives and obstetricians in secondary and tertiary care are autonomous professionals. Nevertheless, they need to coordinate activities to support women during their pregnancy and labor/birth such as sharing information related to pregnant women. This is especially necessary when pregnant women transfer from one level of care to the other. As professionals in the three levels of care are autonomous and yet *inter*dependent on each other in order to deliver optimal care, the Dutch midwifery and obstetric system is imbued with inherent coordination challenges. In this study, we use Faraj & Xiao’s definition of coordination: “(..) coordination is about the integration of organizational work under conditions of task interdependence and uncertainty” [1].

The current public debate in the Netherlands, along with two key public reports, emphasizes the need for improved coordination in midwifery and obstetrics, especially between primary and secondary care [2,3]. However, coordination has not yet been systematically studied in this sector. We aim to fill this gap by conducting a field study on coordination challenges within primary and secondary care in the region of Rotterdam, the second largest city of the Netherlands. By interviewing and observing caregivers, we investigated which factors are frequently mentioned as barriers to successful coordination. This study focuses on the antenatal phase of care as care of women during labor/birth and the postnatal phases could manifest different coordination challenges. In line with our above mentioned definition of coordination, we adopted a practice-based method in order to explore coordination “as it occurs in practice” during everyday working routines [1].

The challenge of coordination is not unique to Dutch midwifery and obstetrics and is also present in other health care sectors in the Netherlands and abroad, where professionals are highly specialized. Specialization allows professions to develop their own expertise, but also makes it more difficult to then integrate their various contributions in order to deliver optimal care [4]. Although recently changes have been made to medical education, many professionals were still educated to believe that the quality of their provided care depends on their individual knowledge and hard work and not on coordination with others [5]. As such, it is not surprising that health care is viewed as particularly burdened by the coordination challenge [6].

There are two major perspectives on how coordination can be achieved. The organizational design-perspective is the traditional perspective, which argues that it is possible to achieve optimal coordination with the right *organizational structures* in place, such as rules and protocols [7]. More recent studies point out that this assumes a static and predictable environment of an organization [1]. Emphasizing that many organizations work in dynamic environments and are faced with time constraints and uncertainty, Faraj and Xiao argue for a coordination-practice perspective [1]. These studies point to the importance of *interprofessional communication* (in addition to organizational structures) to deal with an unpredictable environment [8]. In line with this perspective, Gittell has developed the ‘Relational Coordination Theory’, highlighting that coordination is a fundamentally *relational* process [9].

### The Dutch midwifery and obstetric care system

Community midwives care for women estimated to be at ‘low-risk’ for obstetric and medical complications from the early antenatal until the postpartum period. If women remain low risk throughout pregnancy, women have the option of birthing at home, at a birthing centre (community midwife-led centre in proximity of hospital) or in a hospital, in all cases under the supervision of their community midwife. In 2012, 84.7% of pregnant women started antenatal care with a visit to a community midwife. At the onset of labor, 51.6% of women were still under the care of their community midwife [10]. As such, community midwives play a key role in the provision of maternal health care in the Netherlands.

Should complications (threaten to) occur, community midwives refer women to secondary care in a hospital setting [11]. If necessary, obstetricians refer women with very high maternal or fetal risk to tertiary perinatal care, which is located in eight academic hospitals and two additional non-academic hospitals with obstetric high care and neonatal intensive care units. In 2012, 15.3% of women entered antenatal care in secondary or tertiary care due to their high-risk medical and/or obstetric history [10]. Secondary and tertiary care is provided by obstetricians, resident obstetricians and in most hospitals also by hospital-based midwives (midwives specifically trained to work in a clinical setting) [12].

### Coordination and performance outcomes

Several studies in health care have found a relationship between coordination and performance outcomes in the area of efficiency (e.g. length of hospitalized stay, costs) and effectiveness (e.g. patient satisfaction, clinical outcomes) [9,13]. It would be particularly relevant to improve coordination in the Dutch midwifery and obstetrics as perinatal mortality rates are still relatively high compared to other European countries, and also unequally distributed across neighborhoods [14,15]. In 2010, the extended perinatal mortality rate (deaths from 22 weeks of gestation up to 28 days postpartum) was 9.0 per 1000 births [16]. In socio-economically deprived neighborhoods of the four largest cities, perinatal mortality can be over 30 per 1000 births [15].

## Methods

### Gathering data

We conducted a field study consisting of interviews and observations in order to investigate coordination within midwifery and obstetrics in the region of Rotterdam. The data collection took place in the summer of 2012.

The decision to opt for a qualitative design was based on two arguments. First, the qualitative approach allows to inductively explore the current factors that make it challenging to achieve coordination in Dutch midwifery and obstetrics. Second, asking “how” questions rather than “how many” allowed us to gain a richer and deeper understanding of our field site. Whilst the results of this study cannot be generalized to a larger or different population, they do indicate how coordination can be improved in Rotterdam.

#### ***Selection***

The selection of informants was done by purposive sampling. This means that we chose respondents based on specific characteristics to ensure the inclusion of a wide range of perspectives. We included community midwives, hospital-based midwives, obstetricians and resident obstetricians. All obstetric departments of all hospitals and all midwifery practices in the region of Rotterdam were contacted and invited to participate. We spoke to at least one hospital-based midwife and two obstetricians from each of the seven hospitals in the region of Rotterdam (excluding one hospital which does not employ hospital-based midwives). We interviewed community midwives from 13 out of the 33 midwifery practices in the region of Rotterdam. When scheduling interviews with community midwives, we attempted to interview caregivers located in diverse neighborhoods, ranging from urban to more rural, and high-income to deprived neighborhoods.

#### ***Interviews***

We conducted 40 interviews with 13 community midwives, 8 hospital-based midwives and 19 obstetricians (including two resident obstetricians). We interviewed caregivers from a tertiary hospital, which also acts as a secondary care hospital and as such works together with community midwives (to protect anonymity this hospital is referred to as belonging to secondary care from this point onwards). The first and second author (a social scientist and a non-practicing medical doctor, respectively) conducted most of the interviews, with additional support from two social scientists. The interviews were semi-structured and consisted of broad and open questions (see Additional file 1 in the appendix). We asked questions regarding coordination experiences, the perceived consequences of misaligned coordination, and how caregivers dealt with coordination challenges.

#### ***Observations***

To complement the interviews and further enhance the quality of the data, the first author conducted non-participatory observations. Each of the four types of professionals was shadowed during a typical workday, which included interaction with pregnant women. In the hospital settings, this included the outpatient clinic and consults between (resident) obstetricians and community midwives. A community midwife was shadowed during regular consulting hours. These observations took place at three different hospitals and one midwifery practice. The observed care providers were all individuals whom we had interviewed beforehand. During and at the end of the day, they were willing to answer questions that arose during the observations. Next to this, three multidisciplinary meetings to discuss the organization of care as well as a perinatal audit meeting discussing substandard care led by midwifery and obstetric professionals were observed. The four studied types of caregivers were present at all of these meetings.

#### ***Role of researchers and consent***

The first and second authors are affiliated to a tertiary medical center. As such, the researchers worked in the same department as a few of the respondents. The researchers did not know the large majority of the other respondents outside of the department. All of the interviews were audio recorded and the contents as well as the field-notes were fully transcribed without any identifying characteristics of the respondents. Consent was obtained from all observed and interviewed caregivers. We do not reveal any confidential or potentially identifying data of care providers and pregnant women. During the observations, it was the responsibility of the shadowed care providers to clarify the presence of the researcher and ask pregnant women for consent. In this study we do not include any data from pregnant women who did not provide consent. This study was exempt from an ethical approval in the Netherlands as it did not require respondents to take any specific actions (such as taking blood tests). For more information, please see the Dutch CCMO (Dutch Central Committee on Research Involving Human Subjects) website: http://www.ccmo.nl/nl/uw-onderzoek-wmo-plichtig-of-niet nl^a^.

### Analysis

The analysis of the interview transcripts and observation field-notes was conducted to identify coordination challenges. We used directed content analysis in order to create codes for the analysis. This means that key concepts derived from existing literature are used to form preset codes. Directed content analysis was chosen as it is suitable when trying to support existing theoretical frameworks, or when applied to a novel context [17]. We used key concepts of the organizational-design perspective to identify codes for organizational structures. Examples of these preset codes are ‘obstetric protocols’ and ‘midwifery guidelines’ [8]. Sketching the organizational structures can help to understand the context within which coordination practices occur. Drawing on research from the coordination-practice perspective, we used Gittell’s relational coordination theory to derive codes for interprofessional communication, such as ‘mutual respect’ or ‘frequency of contact’ [8,9].

During the coding process of the first eight interviews, we also used emergent codes in order to facilitate a possible extension of the existing literature. The customized coding list (containing preset and emergent codes) was used to analyze the remaining interviews and field-notes. All analyses were done using ATLAS.ti 7. To increase the trustworthiness of our interpretation of the data, we reviewed the preset and emerging concepts with midwifery and obstetrics providers during both the fieldwork and the analysis phase. The fourth author, a non-practicing community midwife and a colleague of the first author, a gynecologist, also provided valuable feedback as experts from the field regarding whether the codes adequately represented the empirical data. All of the quotes used were translated into English by an English native speaker, and then translated back into Dutch by a Dutch native speaker to check for consistency. The analysis performed on the data collected allowed us to identify patterns of coordination in midwifery and obstetrics in the Netherlands. We paid attention to both the respondents’ perception of coordination, and the actual coordination routines as we saw them unfold.

## Results

**Figure 1:**
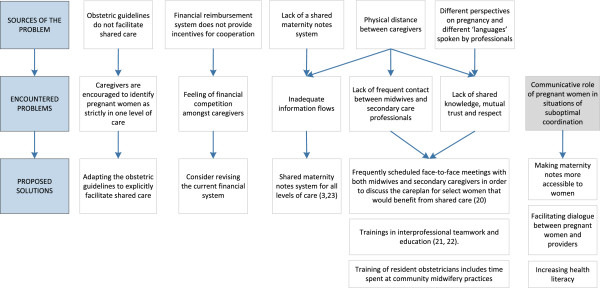
**Sources of the coordination problems, encountered problems and possible solutions. **This figure summarizes the identified sources of a given problem and the encountered problems, based on the findings of this study. Moreover, it indicates possible solutions suggested by the authors. It should be noted that the currently encountered problems (middle row in the figure) are seen as contributing to the problem of ‘communicative role of pregnant women in situations of suboptimal coordination (far right in the figure). The arrows indicating this have not been added in order to reduce figure complexity.

We found that all caregivers interviewed mentioned a variety of factors they currently employ to facilitate coordination. Most frequently cited were multidisciplinary meetings in ‘collaborations in midwifery and obstetrics’ (*verloskundige samenwerkingsverbanden*), which allow for deliberation between community midwives and obstetrical caregivers regarding the organization of care and the care for specific pregnant women. In order to indicate areas for improvement, we focus on commonly cited and observed unmet coordination challenges. Figure 1 (see ‘discussion’) provides an overview of the most commonly identified problems. For an overview of the number of respondents who mentioned these specific problems, see Additional file 2 in the appendix.

The results indicate that the current system of midwifery and obstetric care makes it challenging for community midwives and secondary obstetric caregivers to achieve coordination. As an obstetrician explained: “These two systems [of care], they don't understand each other”. Coordination problems mostly emerged during referral from one level of care to another level. According to national data, these referrals occur frequently in the Netherlands: in 2012, approximately 32.9% of women who started care at the primary level switched to the secondary or tertiary level of care before the onset of labor.

The current ***organizational structures*** seem to separate community midwives and secondary caregivers and often do not encourage joint deliberation. For one, the current obstetric guidelines classify women into one level of care. They do not arrange for shared care, where a pregnant woman could be, for instance, seen by both a community midwife and an obstetrician. The obstetric guidelines do leave room for deliberation between the levels of care, but this is primarily employed to decide which level of care a pregnant woman belongs to.

Next to these guidelines, there is also a clear physical separation between community midwives and secondary caregivers, as community midwifery practices are mostly located in neighborhoods, away from hospitals. As such, formal and informal contact between primary and secondary caregivers typically does not take place on a daily basis during the antenatal phase of care. Moreover, community midwifery practices and hospitals use different and non-compatible maternity notes (also referred to as antenatal notes or patient files) systems. The process that most hospitals and community midwifery practices use for exchanging information relating to pregnant women involves several steps Community midwives print out a summary of their maternity notes and ask the pregnant woman to hand this to the secondary caregiver. This document is then scanned and added to the hospital maternity notes. Should a pregnant woman move back to the community midwife, secondary care-providers update the responsible community midwife via telephone, fax, email or post, by providing a summary of their maternity notes.

Moreover, community midwifery practices and most secondary hospitals are financially autonomous, which means that their income partially depends on the number of women in their care, and the type of care provided. Caregivers explained that this could lead to an incentive not to refer women to other caregivers. Without exception, all caregivers stated that this created an unwanted situation of competition and discouraged collaboration.

Different ***perspectives*** on antenatal health also seem to separate community midwives and secondary caregivers. An insightful illustration of this is that community midwives refer to pregnant women as ‘clients’ and secondary caregivers use the term ‘patients’. Community midwives emphasized that pregnancies are a fundamentally physiological process. As an obstetrician observed, this made some community midwives reluctant to collaborate with secondary care:

“I think that [community midwives] are definitely in support of working with secondary care, but for now the perceived threat that pregnant women will be medicalized is way too big, this clashes with their ideas of a physiological birth”.

Some secondary caregivers also reported that they felt that they did not speak the same ‘language’ as community midwives and therefore did not always understand each other. On the basis of our interviews and observations, we found that obstetric caregivers tend to use more ‘medical’ terms to convey the same meaning. Several obstetricians explained that frequent contact with community midwives in multidisciplinary meetings (‘obstetric collaborations’) helped to overcome the feelings of frustration resulting from different perspectives on antenatal health.

The current state of ***interprofessional communication*** also hinders the achievement of coordination in Dutch midwifery and obstetrics. For one, we found that *shared knowledge* between primary and secondary care-providers was partially missing. All community midwives reported being somewhat familiar with what secondary obstetric caregivers do. Hospital-based midwives who used to be community midwives were highly knowledgeable about both ‘worlds’. However, many (resident) obstetricians stated that they were largely unaware of what community midwives actually do, including how they screen for risks. This also became apparent during our observations. In addition, almost all caregivers stated that there was *inaccurate communication* during referrals and consults, where essential information related to pregnant women was not referred correctly and/or completely, or not transferred at all.

All caregivers mentioned *mutual respect and trust* between community midwives and obstetricians. The issue of respect was particularly emphasized by community midwives, and commonly associated with a perceived hierarchy. Frequently mentioned issues were: obstetricians not taking the medical opinion of midwifes seriously, a lack of trust between community midwives and obstetricians and a feeling of being in competition with each other. We also found that the abovementioned elements - fragmented organizational structures, different perspectives on antenatal health and problematic interprofessional communication - are intertwined. This is illustrated by the following situation, where not seeing how other professions work due to infrequent face-to-face contact was intertwined with a lack of shared knowledge of each other’s policies and consequently not trusting the other professional. A community midwife reported that when she transferred a client to a specific hospital, the secondary caregivers always re-ordered the laboratory blood measurements, even when she had sent them the results of blood tests she had recently ordered herself. She felt that this was a sign of lack of trust in community midwives, and that she did not want to work with the hospital anymore. However, interviews with obstetricians from this very hospital revealed that it was hospital policy to always re-order blood measurements from any external care unit. The community midwife was not aware of this hospital policy.

### Pregnant women as communicators

We found that pregnant women at times needed to compensate for suboptimal coordination between community midwives and secondary caregivers. As already indicated above, one major area of suboptimal coordination is the transmission of information related to pregnant women between midwives and secondary care professionals. Pregnant women who were referred between primary and secondary care sometimes forgot to take a hardcopy of their maternity notes with them. When this happened, professionals did not have immediate access to these notes due to the lack of a shared digital maternity notes system in Dutch obstetrics and midwifery. Even when the maternity notes were transferred correctly between primary and secondary care, the contained information was not always accurate. During our observations and based on the interviews, we found that professionals frequently dealt with these coordination problems by asking pregnant women to provide information about the care received at the other care level, and sometimes the results of relevant tests. These questions went beyond the standard intake questions that are routinely asked after referral. During our observations, and based on the perception of the interviewed professionals, some women had difficulty answering these questions – especially regarding the specific results of tests that had been done.

Based on our interviews, women not only transmitted information, but also needed to correct or add information in the process of referral from one level of care to the other. For example, a woman had had a previous child with a metabolic disease. This information was known to the community midwife, but not conveyed to the obstetrician who later on became responsible for the care of the woman. The obstetrician only discovered the history of metabolic disease because the pregnant woman had mentioned it.

## Discussion

Our research indicates that community midwives and secondary obstetric professionals at times work in fragmented worlds. This fragmentation can be understood from an organizational-design perspective, as we identified problematic organizational structures, such a lack of a shared maternity notes system and misaligned financial incentives. Additionally, in line with the more recent studies taking a coordination-practice angle, the results show that there were also a number of coordination practices that made coordination difficult. Important were different perspectives on antenatal health and suboptimal interprofessional communication. Thus organizational structures and coordination practices hindered caregivers in achieving optimal coordination. These challenges also exist outside of Dutch midwifery and obstetrics, and have been shown to have adverse effects on organizational efficiency and effectiveness [1,8,18].

An unexpected finding of this study is the communicative role of pregnant women in support of interprofessional coordination. Pregnant women played a role in transferring and correcting information between community midwives and secondary caregivers. This is an outcome that none of the caregivers in our study aimed for, but seems to be the result of a number of currently suboptimal coordination practices, as outlined in this article.

As pregnant women support coordination between community midwives and secondary caregivers, they may be experiencing tensions similar to ‘boundary spanners’ [19]. Pregnant women who are able to effectively communicate might help facilitate coordination between separate organizations. However, we expect that women who are less educated and/or not fluent in Dutch have more difficulties fulfilling this communicative function. Therefore, these women might be particularly disadvantaged. This may contribute to the existing perinatal health disparities associated with socio-economic status in the Netherlands (see introduction).

Having pregnant women take on a communicative role in situations where they might not fully be able to do so is not only problematic in the setting of Dutch midwifery and obstetrics, but in the entire health care sector. Health care professionals are highly specialized, and clients are typically without expert knowledge. In the case of Dutch midwifery and obstetrics, this imbalance in expertise makes it very challenging for pregnant women to understand and accurately engage with the information received from caregivers and navigate through oftentimes complex and fragmented systems of care.

The communicative role of clients/patients is a central theme in the field of ‘patient participation’, which is expected (but thus far rarely proven to) increase quality of care, care outcomes and ultimately, patient empowerment [20-22]. However, it does not seem that the findings of our study are examples of patient participation. At the lower end of the patient empowerment scale, participation is seen as informing pregnant women so that they are able to join in discussions about their condition. At the higher end of this scale, participation is conceptualized as enabling clients/patients to join in the decision-making process [21]. The findings in our study did not include joint decision-making. Pregnant women did not *receive* information for the purpose of greater participation, but actually they were *transmitting* information in situations where there was presumably an expertise gap between them and the professional.

Next to studies on patient participation, studies on coordination focus on the *effect* of coordination on clients, such as on patient satisfaction or clinical outcomes [9,13]. For instance, Gittell’s model of relational coordination is increasingly used to assess coordination practices, but it does not include a possible communicative role of the client [9]. As such, the coordination literature currently treats clients as merely recipients, rather than as supporters or even as co-producers of coordination. This study indicates that research on coordination should incorporate the experiences of clients. The term ‘stake-holder coordination’ would be more apt in incorporating the role of clients in coordination processes than the currently used term ‘interprofessional coordination’.

### Practical implications

We found that pregnant women are at times required to take on a communicative role to facilitate coordination. This might be an additional indication for the need - already felt by the professionals - to improve coordination in Dutch midwifery and obstetrics. Fortunately, a large number of initiatives are currently in place to improve coordination in Dutch midwifery and obstetrics. Based on the results of this study, we recommend a number of measures that could help improve interprofessional coordination and thereby minimize the necessity for pregnant women to take on a communicative role in support of coordination, as outlined in Figure 1.

We recommend more frequently scheduled face-to-face meetings with both midwives and hospital-based caregivers in order to discuss and improve coordination practices as well as the care pathways for women that would benefit from shared care, i.e. the involvement of more than one level of care [23]. Such meetings are already in place in some areas and could increase interprofessional communication, such as mutual trust and shared knowledge [18]. This could concurrently be achieved by implementing training in interprofessional teamwork and education [24,25]. In terms of education, we recommend that the training of resident obstetricians include time spent at community midwifery practices. Moreover, we support the current movements in the Netherlands towards a shared maternity notes system for all levels of care, as is in use in part of the UK [3,26].

A concurrent strategy would be to improve the communicative capacity of pregnant women so they are better equipped to support interprofessional coordination, if they need to. This could be done by exploring ways of making provider-information more accessible to pregnant women, facilitating more dialogue between pregnant women and providers, and increasing health literacy. Although effectiveness studies remain scarce [20], some potentially interesting interventions exist, such as http://www.mijnzorgnet.nl^b^ a website that allows clients, their social network and providers to share and discuss health-related information. However, it should be noted that increasing the communicative capacity must only be seen as a potential complimentary strategy. Pregnant women cannot be expected to master the technical knowledge in order to fully navigate the midwifery and obstetrics system and the prevailing medical expertise.

### Limitations & future research

While we conducted a relatively large number of interviews, we only spent several days doing observations of obstetric practices, which is brief compared to traditional standards. The scope of this study was the region of Rotterdam. This was done in order to provide a detailed picture of local coordination challenges. Whilst the results cannot be generalized, we believe that they do indicate possible areas in need for improvement in midwifery and obstetrics in the entire Rotterdam region and in other regions in the Netherlands. This is because almost all of these regions have autonomous yet interdependent primary and secondary care systems; and the organizational structures that complicate coordination in Rotterdam such as lack of a shared maternity notes and physical distance can also be found elsewhere in the country [3].

We recommend extending the scope of coordination studies to include a broader range of coordinating stakeholders. This could be done by studying the coordinating roles of other professionals, such as nurses, general practitioners and managers. Moreover, it would be interesting to conduct interviews and more observation moments with pregnant women themselves in order to better understand the role they play within coordination processes. Lastly, it would be interesting to conduct studies on the role of clients in the coordination process in other health care sectors.

## Conclusions

This study indicated coordination challenges within Dutch midwifery and obstetrics in the realm of organizational structures, perspectives on antenatal health, and interprofessional communication. An unexpected finding of this study is that some pregnant women played an active role in communicating in situations of suboptimal interprofessional coordination. We argue that these findings underline the urgency to improve coordination. We recommend increasing multidisciplinary meetings and training, revising the financial reimbursement system, implementing a shared maternity notes system and decreasing the expertise gap between providers and clients. Moreover, monitoring the manner in which clients actively communicate due to imbalances in the coordination of care should garner more attention in future research.

### Endnotes

^a^http://www.ccmo.nl/nl/uw-onderzoek-wmo-plichtig-of-niet

^b^https://www.mijnzorgnet.nl/; accessed on 2/05/2013

## Competing interests

The authors declare that they have no competing interests.

## Authors’ contributions

VLNS conceived of the study and its design, coordinated and helped conduct the interviews and all of the observations, analyzed the data and drafted the manuscript. APG helped coordinating data collection and conducting the interviews and revised the draft manuscript for intellectual content. HG helped interpreting the data and revised the draft manuscript for intellectual content. AJWP participated in the study design and helped to draft the manuscript. PG participated in the study conception and design and helped draft the manuscript. SD participated in the study conception and design and helped draft the manuscript. All authors read and approved the final manuscript.

## Pre-publication history

The pre-publication history for this paper can be accessed here:

http://www.biomedcentral.com/1471-2393/14/145/prepub

## Supplementary Material

Additional file 1 Interview protocol. Click here for file

Additional file 2 Most frequently identified coordination problems by caregivers. Click here for file
