# Supplementary material for: Improving interprofessional coordination in Dutch midwifery and obstetrics: a qualitative study
Source: BMC Pregnancy Childbirth. 2014 Apr 15;14:145. doi: 10.1186/1471-2393-14-145 (PMC4021099; doi:10.1186/1471-2393-14-145)
Supplement: Additional file 1 — Interview protocol. [file 1471-2393-14-145-S1.docx]

APPENDIX I

**Interview Protocol**

*We would like to ask you for permission to record the interviews with a voice-recorder. This recording will help us to transcribe the conversation, after this the recording will be deleted immediately. We will not reveal any confidential or potentially identifying data of you.*

1. Can you provide a concrete example of a situation where you were involved and where working together with [primary/secondary care providers] went well?

2. Can you provide a concrete example of a situation where you were involved and where working together with [primary/secondary care providers] did not go well?

3. What is your definition of adequate interprofessional collaboration in midwifery and obstetrics?

- In which ways would you like to organize interprofessional collaboration?

- In your opinion, how can support for interprofessional collaboration be improved?

4. How is responsibility over pregnant women organized when a woman is in contact with both primary and secondary care givers?

- Could this situation be improved, and if yes, how?

5. Does your obstetric collaboration (formed by a hospital and associated community midwifery practices) have a shared patient file?

- If not, how is communication between the levels of care arranged, and how is the transferral of from one level of care to the other organized?

- What is your opinion about how this is currently done?

6. How could one make sure that there is room for change and improvement within primary and secondary care?

7. At this moment, how do you experience the collaboration between community midwives and obstetricians in the Netherlands/ your region / at your own work practice?

- What are the most important causes of possible problems in working together?

- How could these causes be overcome?

- Do you think that your attitude towards and experience with [primary or secondary care givers] plays a role in working with them?

- When would or do you feel that interprofessional collaboration is really successful?

8. Do you have any further comments, or suggestions?

*NB: A number of questions from the interview protocol were omitted here as they go beyond the scope of this paper. Answers to these questions were excluded from the analysis.*
