# Supplementary material for: Improving interprofessional coordination in Dutch midwifery and obstetrics: a qualitative study
Source: BMC Pregnancy Childbirth. 2014 Apr 15;14:145. doi: 10.1186/1471-2393-14-145 (PMC4021099; doi:10.1186/1471-2393-14-145)
Supplement: Additional file 2 — Most frequently identified coordination problems by caregivers. [file 1471-2393-14-145-S2.docx]

APPENDIX II

**Most frequently identified coordination problems by caregivers**

| **Identified problem** | **# Respondents that mentioned this as a problem - total:**  8 hospital-based midwives (HBM),  19 obstetricians (OB)  13 community midwives (CM) |
| --- | --- |
| Inadequate information flows due to lack of shared maternity notes system | ALL |
| Lack of frequent contact | HBM 5  OB 9  CM 11 |
| Caregivers are encouraged to identify pregnant women as strictly in primary or strictly in secondary care | Observation, not in interview transcripts |
| Feeling that financial incentives creates competition amongst caregivers | ALL |
| The different perspectives on pregnancy and different ‘languages’ spoken seem to create tensions | HBM 4  OB 10  CM 8 |
| Reluctance of community midwives to work together with obstetric caregivers due to fear of medicalization | HBM 3  OB 7  CM 9 |
| Lack of shared knowledge, especially between community midwives and obstetricians | HBM 7  OB 7  CM 11 |
| Inaccurate communication during transferals and consults, leading to inefficiency and suboptimal care | HBM 8  OB 18  CM 11 |
| Problematic mutual respect and trust between primary and secondary care | ALL |
| Pregnant women transferring information | HBM 4  OB 9  CM 8 |
| Pregnant women correcting and integrating information flows | OB 9  CM 3 |
| Pregnant women coordinating provision of care | HBM I  OB 6  CN 6 |

Table showing the most frequently identified coordination problems by all interviewed professionals (8 hospital-based midwives (HBM), 19 obstetricians (OB) and 13 community midwives (CM).
